# The role of hospital environment in transmissions of multidrug-resistant gram-negative organisms

**DOI:** 10.1186/s13756-020-0685-1

**Published:** 2020-02-11

**Authors:** Po Ying Chia, Sharmila Sengupta, Anjanna Kukreja, Sasheela S.L. Ponnampalavanar, Oon Tek Ng, Kalisvar Marimuthu

**Affiliations:** 1 National Centre for Infectious Diseases, Jln Tan Tock Seng, Singapore; 2 Department of Infectious Diseases, Tan Tock Seng Hospital, Block H, CHI Level 3, 18, Jalan Tan Tock Seng, 308442 Singapore; 3 Lee Kong Chian School of Medicine, Nanyang Technological University, Nanyang, Singapore; 4 University Malaya Medical Centre, Kuala Lumpur, Malaysia; 5 Yong Loo Lin School of Medicine, National University of Singapore, Kent Ridge Rd, Singapore

**Keywords:** Multidrug-resistant gram-negative organisms, Environment, Transmission, CRE, CRAB, Carbapenem-resistant *Acinetobacter baumannii*

## Abstract

Infections by multidrug-resistant (MDR) Gram-negative organisms (GN) are associated with a high mortality rate and present an increasing challenge to the healthcare system worldwide. In recent years, increasing evidence supports the association between the healthcare environment and transmission of MDRGN to patients and healthcare workers. To better understand the role of the environment in transmission and acquisition of MDRGN, we conducted a utilitarian review based on literature published from 2014 until 2019.

## Introduction

Multidrug-resistant (MDR) Gram-negative (GN) organisms (MDRGN), specifically carbapenem-resistant (CR) organisms, are a recognized healthcare problem worldwide [1]. Various mechanisms are involved in the development of carbapenem resistance depending on the bacterial species. However, since their discovery, carbapenemases (e.g., NDM, KPC, and OXA) have emerged as key drivers of carbapenem resistance across various Gram-negative bacterial species [2, 3]. Lack of effective treatment and the consequent high mortality [4] has increased emphasis on the prevention of MDRGN transmission. Prevention toolkits and guidelines have been drawn up by various organizations to harness the principles of infection control and prevention to break the chain of transmission and control the spread of MDRGN [5–7].

**Table 1:** Selected papers describing the transmission of multidrug-resistant Gram-negative organisms from the environment to the patient

Sites	Organisms	Molecular methods	Number of Samples	Time	Setting	Ref
Aqueous environment						
Sinks and ultra-filtrate bags	Multidrug-resistant *Pseudomonas aeruginosa*	Repetitive element palindromic polymerase chain reaction (Rep-PCR)	21 unique patients Environmental samples from 5 sinks	Jan 2012 – February 2014	Intensive care unit	[18]
Plumbing (Shower drains, sink taps, sink drain tailpieces, sink drain strainers, sink trap water, toilet bowls)	Carbapenem-resistant Enterobacteriaceae	Whole Genome Sequencing single nucleotide variation	268 clinical isolates 927 environmental samples	April 2014–December 2014	General wards	[21]
Sink faucet aerators	Carbapenem-resistant *Pseudomonas aeruginosa*	Amplified Fragment Length Polymorphisms	46 unique patients 236 environmental samples	February 2009 – January 2012	Intensive care unit	[22]
Sink drains (drainage pipe in the wall)	Metallo-β-lactamase-producing *Pseudomonas aeruginosa*	Pulsed-field gel electrophoresis	14 unique patients Environmental samples from 12 sink drain in patients’ rooms	2008–2014	General wards	[23]
Sink faucets, water samples (Hospital plumbing infrastructure)	Multidrug-resistant *Sphingomonas koreensis*	Whole Genome Sequencing Average nucleotide identity	12 unique patients Environmental samples (Sinks, faucets, pipes, valves, aerators, other plumbing fixtures)	2006–2016	Intensive care unit and General wards	[24]
Dry Environmental Surfaces						
Bedside	Extensively drug-resistant *Acinetobacter baumannii*	Rep-PCR	8 unique patient samples An unknown number of non-clinical samples	March–May 2014	Intensive care unit	[36]
Patient Environment	OXA-48-Enterobacteriaceae MDR *Acinetobacter baumannii*	Clone specific PCR	13 OXA-48 producing Enterobacteriaceae 18 MDR *A. baumannii* An unknown number of non-clinical samples	July–October 2015	Intensive care unit	[37]
Medical equipment and other appliances						
Velcro of blood pressure cuffs	Carbapenem-resistant *Acinetobacter baumanii* (CRAB)	Pulsed-field gel electrophoresis	20 unique patient samples First round: 222 environmental samples Second round: 97 environmental samples (including 2 blood pressure cuffs velcro and 13 healthcare workers)	December 2011 – April 2012	Intensive care unit	[48]
Positioning pillow	Carbapenem-resistant *Klebsiella pneumoniae*	Whole Genome Sequencing, Multilocus Sequence Typing (rpoB, gapA, mdh, pgi, phoE, infB and tonB)	89 unique patient samples, 1030 environmental samples, 24 miscellaneous samples (animals, surrounding hospitals, other countries)	July 2010 – April 2013	Intensive care unit and general wards	[49]
Ice machine	Carbapenem-resistant *Acinetobacter baumannii*	Multilocus Sequence Typing, Rep-PCR	20 unique patient samples, ~ 75 non-clinical samples (50 environmental, ~ 10 from 2 ice machines, 15 HCWs)	September 2015	Spinal Cord Unit	[50]
Cold tea dispenser	Carbapenem-resistant Enterobacteriaceae	Whole Genome Sequencing, Multilocus Sequence Typing	145 unique patient samples, 195 environmental samples	May 2014 – December 2014	Paediatric wards	[52]

Over the past few years, there has been an increase in reports associating MDRGN’s persistence in the hospital environment and subsequent transmission, which has resulted in a greater emphasis on environmental hygiene. To better understand the role of the environment in the transmission of CR Enterobacteriaceae (CRE), CR *A. baumanii* (CRAB), CR *P. aeruginosa* (CRPA), and other MDRGN, we conducted a utilitarian review based on literature published from 2014 until 2019. We categorised the studies into outbreak and non-outbreak reports and focused on four unique hospital environments: aqueous environment, medical equipment (excluding endoscopes), immediate patient environment, and the air (Table 1).

### Aqueous environment

Hospital plumbing systems are held to stringent standards to reduce transmission of infection to vulnerable patients. However, the aqueous environment presents unique challenges to infection prevention and control (IPC), with wet surfaces providing the solid-liquid interface that predisposes to biofilm formation [8]. These biofilms have been proven to harbour multidrug-resistant Gram-negative organisms (MDRO) [2, 9] which were genetically related to clinical isolates suggesting that aqueous environment can serve as a reservoir for human infections. Furthermore, waste material disposed into the sinks and drains potentially provide the nutrients necessary for the formation and maintenance of biofilms which function as a reservoir for MDRO [10]. These MDRO are not contained within the hospital environment but may spread into the community via the sewage system. Across the world, in Singapore, Bangladesh, India, Lebanon and Spain, raw hospital sewage has been shown to contain Carbapenamase-producing Enterobacteriaceae and other MDRGN [11–17]. As such, it is crucial that environmental contamination and subsequent transmission of MDRGN within the hospital is prevented.

### Outbreak reports

Various plumbing components have been implicated in MDRGN outbreaks including sinks and washbasins in separate outbreaks of CRE, MDR *P. aeruginosa* and polyspecies German imipenemase-1 (GIM-1) [18–21], sink faucet aerators in a CRPA outbreak [22], and multiple plumbing components from the sink drains to wall pipes in a CRPA outbreak [23]. In a CR *Sphingomonas koreensis* outbreak, sink aerators, faucets, mixing valves, pipes and other plumbing fixtures were also found to be contaminated [24]. Shower drains have also been implicated with CRPA outbreak [25].

In a study by Stjärne et al., investigation of a CRPA outbreak revealed contamination of sinks with isolates closely related to CRPA from patients [23]. Following sink replacement, CRPA reappeared on sink surveillance cultures after a mean duration of 13 weeks. Drainpipes were CRPA culture positive as well, suggesting that the reservoir was lower down in the drainage system. Even following acetic acid treatment, 2 wall pipes remained positive after 10 weeks. Sink drains, siphon and pipes to the wall were then changed again, but after 5 weeks, 1 pipe became positive again. All bathroom sinks continued to be treated with acetic acid, and after at least 2 weeks of acetic acid treatment, the nosocomial transmission of CRPA was stopped. Clinical CRPA infections reappeared when the acetic acid treatment was stopped.

An established reservoir of MDRGN deep in the drainage system has also been described in outbreaks involving CR *Sphingomonas koreensis* and KPC-producing *E.coli* [21, 24]. Despite replacing various components of the contaminated sinks for the CR *Sphingomonas koreensis* [24], and extensive replacement of drains and plumbing infrastructure all the way to the central drainage stacks for the KPC-producing *E. coli* [21], the sinks were recolonised after a short period of time. Adjustment of hot-water temperature to 60 °C and augmentation of free chlorine concentrations to at least 0.5 mg per litre resulted in the control of the CR *Sphinogomonas koreensis* outbreak [24]. On the other hand, the KPC-producing *E. coli* [21] persisted at a lower rate of infection in spite of additional control interventions, including cohorting, enhanced cleaning measures, and temporary ward closure for terminal cleaning with sodium hypochlorite, and decontamination with hydrogen peroxide vapour [21].

Retrograde contamination from common sewage pipes can also occur in showers [25]. The use of showers may result in airborne or droplet transmission from shower drains to patients. In a CRPA outbreak study, Hopman et al. demonstrated CRPA in air samples collected immediately and 15 min after running the shower for 10 min. Enhanced infection control and prevention measures were then adopted with daily cleaning and disinfection of environmental surfaces of the patients’ room and washroom. The shower and sink drains were also mechanically cleaned and then disinfected. Surveillance environmental sampling then became negative for CRPA but halting these measures resulted in recontamination within 1 week.

### Non-outbreak reports

In a quasi-experimental study, water safe strategies in the healthcare setting, including removal of sinks from patients’ rooms have demonstrated a decrease in MDRGN infections in an intensive care unit (ICU) setting [26]. Other water safe strategies were also implemented during the study period, including the use of antibacterial water filters which were replaced monthly, replacement of sink siphons and aerators every 3 months, use of filtered water from central sinks and use of 2% chlorhexidine impregnated washcloths for patients’ daily hygiene, and discarding dirty water in a disposal room separate from patient areas. Other infection control and prevention strategies were also introduced, including measures to improve hand hygiene, contact precautions for and cohorting of patients colonized or infected with MDRGN, use of dedicated equipment, updating environmental cleaning protocols and adoption of ultraviolet light disinfection technology for terminal cleaning of isolation rooms. The role of water safe strategies was also supported by another quasi-experimental study in which sink removal and use of water-free patient care in the ICU resulted in the reduction of MDRGN colonisation rates [27]. There was a reduced rate of ICU acquired gram-negative bacilli after removal of sinks and the introduction of water-free patient care. While it is difficult to attribute the reduction of MDRGN to sink removal conclusively, the above studies highlight the potential role of sinks as a source for MDRGN infections and the importance of IPC strategies for prevention of MDRGN acquisition from the aqueous environment.

A surveillance study in an ICU found sink drains located near the toilet were much more likely to be positive for *bla*_KPC_ (20/23 sinks drains) compared to sink drains near the entry door (5/23 sink drains) [28]. The difference in contamination of sink drains based on the proximity to the toilets suggest that sink drains may be contaminated by the droplets generated during flushing of toilets. This emphasizes the need for IPC intervention at the design stage of wards and patient rooms. In a mathematical modelling study by Julia et al. [29], among the risk factors identified for sink contamination were the presence of a sink in adjacent rooms sharing common plumbing, status of sink in the past 30 days, status of patient in the same room, presence of MDRO positive patient in past 14 days, presence of MDRO positive patient in adjacent room, and infection-control interventions performed in past 7 days. The investigators also found that IPC interventions show a non-sustained, negative effect on sink positivity [29]. These findings are in keeping with the aforementioned studies.

Sink basin design, water drainage speed, and connectivity of the plumbing system may potentially influence the occurrence and trajectory of MDRGN outbreaks [30–32]. In an experimental design, replicate hand-washing sinks were inoculated with a green fluorescent protein (GFP)-expressing *E. coli* and dispersal was measured using settle plates and air sampling [30]. In the first 2 weeks of the experiment, no environmental contamination was detected from the sink to the level of the waste trap. However, when a biofilm was allowed to develop, the sink strainer became colonized with GFP expressing *E. coli*. Subsequently, environmental contamination during faucet use occurred as a result of droplet dispersion [30, 31]. Likewise, when the sink bowl was already contaminated, the use of the sink resulted in environmental contamination [30]. These studies also demonstrated the retrograde contamination of separate sink waste traps which shared proximal connections in plumbing.

Sinks could possibly contaminate the immediate surrounding environment for up to 1 m [32]. Key design factors that influenced the environmental contamination were the design of the sink basin, speed of drainage of wastewater, and the location of the sink drains. In an experimental sink model with contaminated sink waste traps, sinks that rapidly drained and or had rear-draining sinks had less environmental contamination [32]. When sink basin drainage was immediately underneath the faucet, environmental contamination occurred regardless of the speed of the drainage but was 8 times greater with slow drainage. The importance of sink basin design was supported by a separate study, where sinks with faucets aligned behind a drain had a higher rate of contamination by *P. aeruginosa* compared to drains aligned directly with, in front of, or to the side of the faucet [33]. Similarly, slower drainage was found to be associated with a higher microbiological burden and drain positivity for contamination [33]. In addition, the presence of a drainage cover was shown to reduce contamination of countertops and healthcare worker (HCW)s’ gowns in a study by Hajar et al. [34]. In the absence of a drain cover, 11% of countertops, 9% of gowns, and 6% of hands after hand washing demonstrated contamination with Gram-negative organisms compared to the contamination of 1% of countertops, 2% of gowns and 0% of hands with drainage cover.

### Summary of evidence and future research

Available observational and quasi-experimental evidence strongly support a significant role of the aqueous environment as a nosocomial reservoir of MDRGN infections both in the outbreak and non-outbreak settings. There is a paucity of randomized controlled trials examining the role of interventions targeting the aqueous environment in reducing rates of MDRGN infections.

Existing evidence also provide a strong mechanistic rationale for potential pathways of dissemination of MDRGN through hospital plumbing and wastewater management systems and subsequent spread to patients. These models suggest that infection risk from aqueous environments can be modified via design changes.

In addition to established infection prevention measures targeting human to human MDRGN transmission, promising environmental interventions demonstrated in quasi-experimental studies to reduce MDRGN infection rates include regular cleaning with acetic acid, water safe strategies, sterilization of water using chemicals or controlled water temperatures and physical replacement or removal of affected plumbing systems. The current evidence is insufficient for conclusive recommendations in international guidelines, including the recently published World health Organizations’ guideline [35], as to the recommended methods for environmental cleaning and disinfection of MDRGN.

There is a pressing need for research examining the effectiveness of aqueous environmental cleaning and disinfection interventions (both individually and as part of bundles) for prevention of transmission of MDRGN. Additional epidemiologic and mechanistic studies examining the factors and pathways affecting the transmission of MDRGN from the aqueous environment would help inform the design of strategies to be tested.

### Dry environmental surfaces

For the purpose of this review, dry environmental surfaces include the immediate patient environment, including high-touch surfaces such as bed rail, bedside tables, and call bells.

### Outbreak reports

Two recent studies examined the role of the physical environmental surfaces in the transmission of MDRO in two separate ICU outbreaks. In an ICU outbreak of extensively drug-resistant (XDR) *A. baumannii* involving eight patients with clinical infections [36], microbiological sampling of the bedside physical environment demonstrated contamination with XDR *A. baumannii*. The outbreak was rapidly controlled after the institution of an intensified IPC bundle which included environmental disinfection, routine disinfection of devices, and terminal cleaning of environment and surfaces. Molecular analysis revealed diversity in the clones of *A. baumannii,* which suggested an ongoing evolution of the isolates and suggests that outbreak strains can quickly adapt over a short time period of 2–3 months [36]. In the second ICU outbreak investigation involving both OXA-48-producing Enterobacteriaceae and MDR *A. baumannii* [37], 13 OXA-48-producing Enterobacteriaceae carriers and 18 MDR *A. baumannii* carriers were identified. Initial outbreak response included in-depth bleach cleaning of the environment and a review of device disinfection protocols. Despite this, environmental surveillance cultures post terminal cleaning showed persistence of OXA-48 on sinks and mattresses. The outbreak was subsequently controlled with a combination of bleach environmental cleaning and hydrogen peroxide vapour.

### Non-outbreak reports

Environmental contamination by MDRGN varies greatly depending on the endemicity, transmissibility, and resistance mechanism of the organisms, sampling techniques, and disinfection protocols. In one of the more extensive studies which was conducted over a period of 32 months, investigators conducted daily environmental sampling for a week and at day 14 or at the point of discharge of patient occupying the room [38]. A total of 2860 samples surrounding the environment of 80 unique patients were obtained. The environment surrounding seventy of these patients demonstrated contamination across all study days [38]. In another study investigating the environmental contamination of CRAB in an endemic setting, investigators found that ICU rooms occupied by patients carrying CRAB were consistently colonized by genomically similar strains of CRAB [39]. The investigators also demonstrated persistence of CRAB in the environment and subsequent clinical infection, highlighting the need for carefully thought out IPC strategies to control MDRO in an endemic setting.

Environmental contamination by colonized or infected patients is a key step in the onward transmission of MDRO, and understanding risk factors for environmental contamination may facilitate preventive IPC strategies. A recent study by Mody et al. [40] demonstrated that contamination of patients’ hands with MDRO is frequent and correlates with contamination of high-touch surfaces. Patients who were colonized or infected with CRAB were also shown to have higher environmental contamination with clonal CRAB compared to patients who were not [39, 41]. The higher burden of MDRGN as usually observed in clinical infections, has been shown to increase environmental contamination [42, 43]. In a study of 26 patients colonized with KPC-producing CRE and environmental contamination, a group of 6 patients were identified as super-spreaders [43]. Majority of these super-spreaders had high rectal CRE concentrations, and faecal incontinence was the only patient-level risk factor for being a super-spreader. Although the median number of CRE colonies found on environmental sampling was 3.5 (IQR 1–11), the 6 patients (18% overall) had more than 50 KPC-producing colonies detected in the environment. The term super-spreader has been used to describe a highly infectious person who transmits an agent of diseases to a disproportionately large number of individuals often via environmental contamination. Lerner et al. in their study of the transmission of KPC-producing CRE identified a group of infected/colonized patients (18%) in whose vicinity the environmental load of the MDRO was high (80%) and classified them as super-spreaders [44]. The status of a super-spreader, however, is not constant. When the rectal concentration of CRE changed, the degree of environmental contamination was also noted to change. These findings stress the importance of early identification and physical separation of MDRO carriers to reduce environmental contamination and prevent onward transmission.

The degree of environmental contamination of dry areas of hospital washrooms, depending on the method of hand drying has also been studied [45]. The 2 methods of hand drying, using paper towels versus using jet air dryers were evaluated. Significantly fewer bacteria, including ESBL-producing organisms, were recovered from the environment when paper towels were used compared to jet air dryers. As such, in high-risk environments, the microbial dispersal risk during the use of medical equipment and para-clinical service equipment needs to be carefully considered and evaluated before adoption and use. In an experiment, test surfaces including over-bed table, different materials including vinyl, stainless steel, Formica, and cloth, were inoculated with CR *K. pneumoniae*, *E. coli* and *Enterobacter* species [46] to evaluate survivability of organisms up to 72 h. Apart from CR *K. pneumoniae* on the Formica surface, all pathogens survived at < 15% at 24 h and all cultures at 72 h were negative [46]. A systematic review by Muller et al. suggested copper surfaces harbour fewer bacteria than non-copper surfaces; however besides an increase in cost, there remains uncertainty about the efficacy for prevention of healthcare-associated infections or MDRO [47].

### Summary of evidence and future research

Studies have demonstrated MDRGN contaminating dry surfaces in the hospital environment can also be responsible for the spread of infection [41]. HCWs responsible for cleaning and decontamination should be educated and monitored for strict adherence to protocols for decontamination of environment where patients infected/colonized with MDRO are housed. Hospital engineers and infection control practitioners need to discuss and collaborate when material choices for environmental surfaces are being made. Coordination between the IC team and Microbiology laboratory on need-based environmental surveillance should be able to pre-empt outbreaks caused by MDRO which are able to remain dormant in the hospital environment.

### Summary of evidence and future research

Majority of the studies on the dry environment were conducted in non-outbreak settings with only two studies implicating the dry environment as a potential source of an outbreak. Most studies were quasi-experimental and observational in nature, potentially because they were conducted as part of routine infection control measures to control the outbreak. Purpose designed studies to address the dry environment’s role in human infections and ways to prevent them are very much needed.

### Medical equipment and other appliances

#### Outbreak reports

Four studies examined the role of medical and para-clinical service equipment in the transmission of MDRGN organisms in an outbreak setting. Equipment implicated in these studies include the Velcro of blood pressure cuffs, positioning pillow, ice machine and a tea dispenser. The Velcro of the BP cuff was implicated in an outbreak of two clones of CRAB among ICU patients [48]. In another report, an outbreak of *bla*_KPC-2_ CR *K. pneumoniae* involving 105 patients in a university hospital was traced back to a positioning pillow and was observed to persist for 21 months [49]. Hospitality equipment may also be a source in an outbreak. One such example would be the discovery of an ice machine as the source of an ongoing CRAB outbreak uncovered incidentally while investigating a CR *K. pneumoniae* outbreak [50]. The investigators demonstrated clonal relatedness of the CRAB isolates from three patients, one HCW, and the ice machine water outlet, which stresses the importance of dedicated IPC strategies for ice machines and other hospitality equipment [51]. In a report from Japan, a cold tea dispenser was also involved in a metallo-β-lactamase (MBL) producing Enterobacteriaceae outbreak [52]. The outbreak which involved diverse species of IMP-1 producing Enterobacteriaceae including *K. pneumoniae*, *E. coli*, *Citrobacter freundii*, *Klebsiella oxytoca* and *Enterobacter aerogenes*, terminated after the removal of the tea dispenser [52].

#### Non-outbreak reports

Studies conducted in non-outbreak settings also suggest portable equipment, personal protective equipment of HCWs, and kitchen cutting boards play a potential role in MDRGN transmission. In an ICU experiment, portable machines were inoculated with a designed DNA marker to investigate the role of portable machines as a vector in microorganism transmission [53]. Doppler ultrasound machines in surgical ICUs and electrocardiogram machines in medical ICUs were inoculated, and high touch surfaces in the patients’ environment, common work areas and other portable equipment were then sampled days after. Results demonstrate contamination of the environment days after inoculation implicating HCWs hands as a possible vector for dissemination of microorganisms in the hospital environment.

Several studies have identified the role of environmental contamination in the transmission of MDRO from the patient to hands and clothes of HCWs leading to further propagation of the organism in the hospital [54–56]. In one study which looked at 254 HCW-patient interactions with 52 patients, A baumannii was identified from HCWs hands or gloves in 30% of the interactions (OR 4.78; 95% CI 1.24–18.45), HCWs touching the bed rail (OR 2.19; 95%CI 1.00–4.82), performing wound dressing (OR 8.35; 95% CI 2.07–33.63) or interacting with the endotracheal tube or tracheostomy site (OR 5.15; 95%CI 2.10–12.60) [56]. In another study, investigators identified other risk factors including positive environmental cultures (OR 4.2; 95% CI 2.7–6.5), time spent in room for a duration of > 5 min (OR 2.0; 95% CI 1.2–3.4), performing physical examinations (OR 1.7; 95% CI 1.2–2.8), and contact with the ventilator (OR 1.8; 95%CI, 1.1–2.8) as crucial risk factors for multidrug-resistant Acinetobacter baumannii (MDRAB) contamination of HCWs protective clothing [54]. Yan Z et al. in their study of 67 new patients with CR-KP found 31.5% of bed units contaminated, 7.9% of positive environmental samples and 3.6% of ICU staff colonized [55].

#### Summary of evidence and future research

These findings stress to importance of identifying index patients of outbreaks in order to confidently investigate the environmental source of outbreak propagation. Compliance to transmission-based precaution, aggressive environmental cleaning, and sustained HCW education in IPC measures may decrease transmission. However, the mechanisms of transmission of MDRGN are not well defined warranting further studies and exploration.

#### Air environment

The role of air transmission of MDRGN is not well established and is inconsistent. Air environment of the patient has been implicated as a possible vector in the spread of MDRO [57]. Biological aerosols can be detected in the air either in the form of nuclei droplets (water or body fluids) or as aggregate microorganisms associated with dry particles. The dry form is likely to be the cause of the spread of healthcare-associated pathogens, including MDRGN [58]. The smaller the particle, the longer they remain suspended in the air and thus get widely distributed, especially in the hospital environment where air movement is uncontrolled. Survival of Gram-negative bacteria in the air depends on bacterial species, particle size and climatic factors such as temperature and humidity. In most studies *Acinetobacter* spp. and *Pseudomonas* spp. have been shown to survive for a considerable period of time [59].

There are two main methods for air sampling, active and passive. Active sampling uses air impactors, centrifugal air machines or filtration systems, which are expensive but require less time. Passive methods are based on sedimentation on settle plates; this requires more time but are least expensive. No one method has shown to be better than the other. Factors which affect sampling are the amount of air contamination (biological burden), type of airflow in the room being tested and activity levels in the room during sample collection [58].

#### Non-outbreak reports

All reported studies were conducted in adult patients and organisms implicated were CRAB and *Pseudomonas* species [60–66]. Majority of the studies were conducted in ICUs [61, 63, 64, 66], one included ICU and step down medical wards [62], and the other was carried out in open wards [60]. In all studies, the air sampling methods differed as some used passive method [61, 63, 65] while others used the active process [60, 62, 64, 66]. Majority of the studies found *A. baumannii*. The results of the studies were mixed, with 5 studies demonstrating air contamination surrounding colonized or infected *A. baumannii* patients and 2 studies showing no association (Thailand [60] and Maryland, USA [64]). The difference seen in these studies could be attributed to the climatic differences, differing IPC practices (close circuit suctioning of ventilated patients), and the difference in methodologies. Regardless, these findings stress the importance of the need for well-designed studies employing state of the art methods to study the role of air environment in MDRO transmission and infection.

Air and environmental surface contamination were significantly higher among rectally colonized patients compared to patients with respiratory colonization (38.3% vs 13.1 and 15.5% vs 5.5% respectively), which was explained by the use of closed-circuit ventilation in an ICU study [65]. Not surprisingly, air closest to patients contained higher concentrations of the organism [66]*.* Preceding activities prior to air sampling also influenced the degree of air contamination with bacterial contamination of greater than 60 times being recorded during treatment activities such as endotracheal suctioning and changing of bed sheets and diapers. However, there was no association between the extent of contamination of air surrounding a patient and burden of CRAB on patients’ respiratory tract or skin [62].

The complex interplay between underlying disease characteristics of the patients and pathogens contaminating the air environment has been documented. For example, in a study conducted by Panagea et al. in a cystic fibrosis treatment facility, *P. aeruginosa* was detected in the majority of air samples collected from patients’ rooms, ward corridors and outpatient clinics [67]. *P. aeruginosa* has also been described to contaminate hospital wards, operation theatres, ICUs and labour rooms [59, 68, 69]. Clonal relatedness between strains isolated from the air and clinical samples show that CRAB can survive in ICU air for approximately 4 weeks, potentially causing further nosocomial infections [66]*.*

#### Summary of evidence and future research

CRAB and *P. aeruginosa* were the most common organisms implicated in the contamination of the air environment in the nosocomial setting. Most of the studies were not designed to show direct transmission of pathogens from the air. Even though the design and conduct of such studies could be resource-intensive, this could be one of the focus of future research. Additionally, more research in identifying the ideal methods for pathogen identification in the air environment is much needed. Future research could include air microbiome analysis, potentially compared with classical microbiological methods, in identifying the role of air environment in persistence and transmission of nosocomial pathogens.

## Conclusions

This review highlights the role of the hospital environment in persistence and onward transmission of MDRGN. Environmental contamination with MDRGN is significant in the outbreak and non-outbreak settings. The aqueous environment seems to be the biggest reservoir for MDRGN in the hospital environment and maybe a source of MDRGN outbreaks and persistence in the endemic setting. This may in part, be due to the difficulties in eradicating MDRGN from the plumbing systems. More research is needed in identifying the optimal IPC strategy to prevent MDRGN transmission from the aqueous environment to patients. Dry environmental surfaces and medical equipment seem to be associated more with Gram-positive and non-fermenting Gram-negative organisms than Enterobacteriaceae [70]. *A. baumanii* was the primary Gram-negative organisms associated with contamination of dry surfaces and subsequent transmission to patients.

Existing reports indicate that, other than direct patient-to-patient transmission and patient-to-HCW-to-patient transmissions, the hospital environment plays a crucial role in the transmission of MDRGN. These reports strongly support the need for a clearly defined IPC strategy to control environmental colonisation and onward transmission of MDRGN. More research is needed to quantify the proportion of MDRGN transmissions via environmental contaminations and identify the most effective IPC strategy to prevent MDRGN transmissions via the aqueous environment.

Further research is needed to quantify the role of the hospital environment in the transmission of MDRGN and IPC strategies to prevent them. Study design should be rigorous and take into consideration the possible influence of climate (tropical, sub-tropical and temperate regions), patient population and ward structure (naturally ventilated and centrally air-conditioned wards). Additionally, the implementation strategy and efficiency of IPC measures may differ according to resource availability, endemicity of MDRGN organisms and the presence of guidelines, especially in low-middle income countries.

## Data Availability

Not applicable.
